# The Effect of Structured Antenatal Education on Childbirth Self-Efficacy

**DOI:** 10.7759/cureus.39285

**Published:** 2023-05-21

**Authors:** Zohour AlSomali, Eman Bajamal, Ola Esheaba

**Affiliations:** 1 Department of Nursing, King Abdullah Medical Complex, Jeddah, SAU; 2 College of Nursing, King Saud bin Abdulaziz University for Health Sciences, King Abdullah International Medical Research Center, Jeddah, SAU; 3 Faculty of Nursing, Alexandria University, Alexandria, EGY

**Keywords:** prenatal education, childbirth self-efficacy, antenatal classes, self-efficacy, antenatal education

## Abstract

Introduction: Antenatal (prenatal) education is a vital role of midwives when giving antenatal care to pregnant women. Particularly in the late stages of pregnancy, antenatal education regarding the natural-labor process, the introduction of labor rooms, coping strategies, and labor-pain management may enhance maternal self-efficacy and perceptions of childbirth. However, educational programs that include birth plans, pain-relief measures, and birth preparation are not a structured part of the Saudi healthcare system. This is the first study to investigate the effect of antenatal education on maternal self-efficacy in Saudi Arabia. The aim of this study was to investigate the effect of an antenatal education program on maternal self-efficacy in primiparous pregnant women and to determine the relationship between maternal self-efficacy and their sociodemographic characteristics in Jeddah, Saudi Arabia.

Methods: A randomized control trial (pretest/posttest) design was conducted with 94 primiparous pregnant women. Two groups were compared: an intervention group, which received a structured antenatal educational program (*n* = 46), and a control group, which received routine antenatal care (*n *= 48). The childbirth self-efficacy inventory (CBSEI) was used to assess maternal self-efficacy. The data were analyzed using IBM SPSS Statistics for Windows, Version 24 (Released 2016; IBM Corp., Armonk, New York, United States).

Results: The mean score on the CBSEI pretest was 238.5 ± 237.4 compared to 242.9 ± 276.2 in the posttest mean score, with significant differences (*p < *.05) in maternal self-efficacy between the pretest and posttest scores for both groups.

Conclusion: The findings of this study suggest that an antenatal educational program could serve as an essential tool, providing access to high-quality information and skills during the antenatal period and significantly enhancing maternal self-efficacy. It is crucial to invest resources to empower and equip pregnant women in ways that promote positive perceptions and boost their confidence regarding childbirth.

## Introduction

Childbirth is a transitional period to parenthood and one of the most challenging experiences in life, with major changes for the expectant mother and her significant other. To ensure that this transitional period is a positive experience, it is essential to prepare the expectant mother with knowledge, information, and coping strategies [1,2]. Recently, the World Health Organization (WHO) has been pushing for antenatal care that facilitates positive pregnancy experiences, which are defined as pregnant women having an effective transition to positive childbirth and achieving positive motherhood that enhances their autonomy, self-esteem, and competence [3]. A positive childbirth experience gives the laboring woman a sense of control through involvement in decision-making during the birth. The midwife has a vital role to play in providing antenatal education and information regarding pregnancy and the birth process [3].

Women are typically required to cope with the childbirth process in labor rooms in unfamiliar environments. This may be associated with low self-efficacy and fear of childbirth, leaving pregnant women doubting their ability to cope with labor [4-6]. Self-efficacy is a cognitive process that enables an individual to evaluate his or her abilities and successfully accomplish designated behavior (efficacy expectancy), while setting her expectations regarding their results when performing a specific task (outcome expectancy) [7]. Lowe (1993) developed the childbirth self-efficacy inventory (CBSEI) to evaluate maternal confidence regarding labor prior to the birth. Lowe conducted a study using the CBSEI to assess the interaction between self-efficacy and fear of childbirth [5]. Researchers suggest that the CBSEI should be used in the antenatal period to identify and support pregnant women with low levels of maternal efficacy. The CBSEI has been used in many studies to examine maternal prior knowledge of the childbirth process and its correlation with maternal confidence regarding childbirth [8,9].

Gagnon and Sandall (2007) conducted a systemic review and concluded that the effect of antenatal education and childbirth preparation is still unknown [10]. However, education programs have been used by many healthcare providers to guide and help pregnant women. Many other researchers have argued that educating women during the antenatal period has a positive impact on pregnancy and childbirth, with reductions in delivery complications, less anxiety, more confidence during labor, and fewer obstetrical interventions [11-13]. In addition, antenatal education improves communication between childbearing women and health care professionals, increases women’s positive labor experiences, decreases the use of analgesics, and provides an opportunity to correct incorrect assumptions regarding the birth process [14].

Khorsandi et al. (2008) proposed that self-efficacy reduces fear and anxiety during the perinatal period. Hence, antenatal education may be an opportunity to enhance pregnant women’s self-efficacy [15]. Furthermore, the interaction between the midwife and the pregnant woman during the antenatal educational classes can build a strong social connection that enhances the woman’s awareness of her own strength and resources. In addition, antenatal educational classes are believed to boost pregnant woman’s confidence in their ability to cope with the childbirth process [16].

Particularly in the late stages of pregnancy, antenatal education regarding the natural-labor process, the introduction of labor rooms, coping strategies, and labor-pain management may decrease fears of childbirth and enhance maternal self-efficacy [17]. In a study conducted in Hamadan, Iran, educational interventions with primiparous women increased the women’s desire for physiological delivery, compared to a control group in the same study [18].

Antenatal educational programs that include birth plans, pain-relief measures, and birth preparation are not a standard part of the Saudi healthcare system. This leaves pregnant women vulnerable to the negative moods that are associated with a lack of or inappropriate information. This study, conducted in Jeddah, Saudi Arabia, investigates the effect of an antenatal education program on maternal self-efficacy in primiparous pregnant women to determine the relationship between their maternal self-efficacy and sociodemographic characteristics. 

## Materials and methods

Design, study participants, and setting

A quantitative, experimental design (randomized control trial, pretest/posttest design) was utilized in this research. The population of primigravida women receiving antenatal care in the study setting was unknown; therefore, the researchers used a non-probability, convenient sampling technique to recruit the participants for this study.

The subjects of the study were low-risk pregnant women who were Arabic speakers and following up and receiving routine antenatal care in a specialized polyclinic affiliated with the Ministry of National Guard Health Affairs (NGHA).

The inclusion criteria for the study subjects were set to ensure homogeneity in the baseline characteristics. The researchers thus recruited primigravida pregnant women in their last trimester, with gestational ages of between 28 and 33 weeks of gestation, with no contraindication for vaginal delivery, such as placenta previa and fetal malpresentation. Multigravida women were excluded from this study, along with women with high-risk pregnancies (e.g., those with gestational diabetes, preeclampsia, etc.) and pregnant women who were not receiving any other antenatal education.

Sample size

All pregnant women who were attending outpatient clinics and met the inclusion criteria were invited to participate in the study. The sample size was estimated by the G*Power 3.1 software program. The sample comprised n = 102 primiparous pregnant women, achieving a power of 80% and an effect size of 0.3, an error probability of 0.05, and a missing data rate of 10%. The estimation was based on a two-tail α of 0.05. The total sample size was n = (92+10) = 102. The participants were divided equally into a control group (n = 51) and an intervention group (n = 51). Of the 102 participants, eight withdrew from the study because of their noncompliance to perform the posttest (n = 94).

Randomization

A sequentially numbered, opaque, sealed envelope (SNOSE) was used. Each envelope contained the assignment information for each participant in the study. The sequence of numbers started from 1 and went up to 102. The intervention and control groups’ information and numbering were divided equally and randomly by a faculty member from the nursing college, who was not included in the study. Half of the envelopes included information on routine antenatal care (for the control group), and the other half contained information on the antenatal educational program (for the intervention group).

Data collection methods, instruments used, and measurements

The study subjects were divided randomly into the intervention group and control group. The control group received routine antenatal care in the clinics. The intervention group received an antenatal education program in addition to the routine antenatal care in the clinic.

Measures

The questionnaire consisted of two parts:

1- Part one requested sociodemographic data whose importance was revealed through a literature search (e.g., age, sex, marital status, residence, monthly income, occupation, level of education, and support system).

2- Part two was the CBSEI. This questionnaire was developed by Lowe (1993) [5] and based on Bandura’s theory of self-efficacy, and it assesses maternal confidence in relation to coping during childbirth. The internal consistency ranges from 0.86 to 0.95. The questionnaire consists of 62 items in two parts. The first section of the instrument measures outcome expectancy and self-efficacy expectancy for active labor. The second part measures the same construct at work. The scale or scores are computed by summing the item responses.

Tool’s validity and reliability

The content validity of the structured interview tool was tested by a group of five experts in the field, and their opinions were taken into consideration. A pilot study was conducted with 10 pregnant women (who were not included in the main study) to assess the clarity, applicability, and comprehensiveness of the tool. The necessary modifications were then applied.

Intervention

The antenatal educational program was provided to the intervention group at 28-32 weeks of gestation. The guide was designed to enhance the pregnant women's self-efficacy and contribute to successful birth experiences. This program was developed by the researchers in accordance with the latest recommendations of the WHO [3] and the Lamaze International Organization (2017) and was reviewed by experts in obstetrics and gynecology. The total duration of the program was five hours and 40 minutes. Each pregnant woman in the intervention group attended two sessions of two hours and 50 minutes each, with a 10-minute break between them. The program was delivered in a well-equipped classroom in a specialized polyclinic affiliated with the Ministry of National Guard Health Affairs, Jeddah.

Statistical analysis

IBM SPSS Statistics for Windows, Version 24 (Released 2016; IBM Corp., Armonk, New York, United States) was used to analyze the data. Descriptive and inferential statistics were also calculated using IBM SPSS Statistics for Windows, Version 24 (Released 2016; IBM Corp., Armonk, New York, United States). The descriptive statistics included the means, standard deviations, frequencies, and percentages of the participant demographics and study variables. Leven’s test was used to compare the baseline characteristics of the intervention and control groups. A table of contingency analysis (cross tabulation) was used to compare frequency distributions based on two variables. In addition, a Chi-square was used to determine whether the cross-tabulated variables had statistical significance. A paired t-test was conducted to identify any difference in the means of the two groups. An analysis of variance (ANOVA) was used to compare the CBSEI pretest and posttest means of the two groups. The Pearson correlation coefficient was used to determine the relationships between two dependent variables at a significance level of 0.05.

Ethical considerations

Institutional Review Board (IRB) approval was obtained from the King Abdullah International Medical Research Center (KAIMRC), along with permission from a specialized polyclinic affiliated with the Ministry of National Guard Health Affairs, Jeddah, Saudi Arabia. The anonymity of the participants was assured by using code numbers to label the questionnaires. After the data-collection phase, the questionnaires were handled only by the researchers and kept in a secure place.

## Results

Table 1 shows the sociodemographic characteristics of the study participants. The participants’ age ranged from 18 to 30 years. As shown in Table 1, the largest age group was 18-25 years (n = 74, 78.7%). All of the study participants (n = 94) were Saudi. Most were living in urban areas (n = 89, 94.7%). Thirty-eight (40.4%) were students, and the majority had undergraduate and/or postgraduate degrees (n = 61, 64.9%). All were earning a monthly income (n = 94, 100%) that was sufficient for their living expenses. In addition, the majority were supported and accompanied by their husbands during the antenatal visit (n = 91, 96.8%).

**Table 1:** Sample Demographic Characteristics (n = 94)

Demographic Variables		n		%	
Age					
	18-25	74	78.7		
	26-30	20	21.3		
Nationality					
	Saudi	94	100		
Residence					
	Urban	89	94.7		
	Rural	4	4.3		
Occupation					
	Housewife	34	36.2		
	Student	38	40.4		
	Employee	22	23.4		
Monthly income					
	Enough	94	100		
Educational level					
	Middle school	7	7.4		
	High school	26	27.7		
	Undergraduate/postgraduate degree	61	64.9		
Attending the clinic with the participants					
	Husband	91	96.8		
	Mother/sister/brother	3	3.2		

Obstetric characteristics of the study participants

As shown in Table 2, most of the participants said that they visited the antenatal clinic regularly (n = 81, 86.2%). The number of antenatal visits attended by the participants (from the beginning of the pregnancy until the time of the study) ranged from 0, if this were their first contact, to 4. Most of the participants (n = 77, 81.9%) had previously attended on three or four occasions. Most said that their primary source of knowledge about labor and birth was social media (n = 60, 63.8%), while 22.3% had obtained knowledge and information from the family and friends.

**Table 2:** Sample Obstetric Characteristics of the Study Participants (n = 94)

Obstetric Variables		n	%
Do you have regular antenatal visit			
	Yes	81	86.2
	No	13	13.8
How many antenatal visits have you attended from the beginning of your pregnancy			
	3-4 visits	77	81.9
	1-2 visits	17	18.1
Primary source knowledge regarding labor and birth			
	Websites	4.0	4.3
	Social media	60	63.8
	Relatives and friends	21	22.3
	Health care provider	9.0	9.6
My prenatal care provider spent time talking with me about my labor and delivery expectations			
	No	94	100
My prenatal care provider prepared me for my birth experience			
	Yes	07	7.4
	No	87	92.6
Have you attended any antenatal/birth educational programs			
	Yes	55	58.5
	No	39	41.5
What is the main antenatal education you received?			
	No antenatal education	31	33
	Antenatal pelvic exercise	21	22.3
	Nutrition during pregnancy	01	1.1
	breastfeeding	41	43.6

None of the participants had spoken with their prenatal care provider regarding their labor and birth expectations, nor had this topic been covered in their discussions during antenatal visits. In addition, the majority (n = 87, 92.6%) said that they had not been prepared for their birth experiences by their health care provider. However, more than half (n = 55, 58.5%) had attended an antenatal educational program, the most common of which concerned breastfeeding (n = 41, 43.6%).

Sociodemographic differences in self-efficacy

Age

As shown in Table 3, 64.8% of women aged between 18 and 25 years reported high self-efficacy (n = 48), with just 1.35% (n = 1) reporting low self-efficacy.

**Table 3:** Relationship Between Sociodemographic and Maternal Self-Efficacy

		Level of Maternal Self-Efficacy			
Sociodemographic Variables		High Efficacy (n)	Moderate Efficacy (n)	Low Efficacy (n)	p
Participant’s age (years)					
	18-25	48	25	1	.413
	26-30	16	4	0	
Residence					
	Urban	59	29	1	
	Rural	4	0	0	.649
	Remote area	1	0	0	
Participant’s Occupation					
	Housewife	29	4	1	
	Student	24	14	0	.021
	Employee	11	11	0	
Wife Educational level					
	Middle school	6	1	0	
	High school	19	6	1	.303
	Undergraduate/postgraduate degree	39	22	0	
Support during antenatal visit					
	Husband	62	28	1	.980
	Mother/sister/brother	2	0	1	

Occupation 

Maternal occupation was significantly associated with childbirth’s self-efficacy (p = .021). Of the total 34 housewives, the majority had high self-efficacy (n = 29, 85.2%) as shown in Table 3.

*Educational Level* 

As shown in Table 3, high self-efficacy was seen among those with undergraduate and/or postgraduate degrees (n = 39, 63.93%).

Obstetric differences in self-efficacy

As shown in Table 4, there was a significant association between a woman’s primary source of knowledge regarding labor and birth and her childbirth self-efficacy (p = 0.047). Low self-efficacy was reported among those participants who obtained their knowledge from relatives and friends (n = 1, 4.76%), high self-efficacy among those who obtained their knowledge through social media (n = 47, 78.33%).

**Table 4:** Relationship Between Obstetric Data and Maternal Self-Efficacy

		Level of Maternal Self-Efficacy			
Obstetric Variables		High Efficacy (n)	Moderate Efficacy (n)	Low efficacy (n)	p
Source of knowledge regarding labor and birth					
	Websites	2	2	0	0.047
	Social media	47	13	0	
	Relatives and friends	12	8	1	
	Health care provider	3	6	0	
My prenatal care provider prepared me for my birth experience					
	Yes	1	0	6	.005
	No	63	1	23	

Antenatal care provider preparation for birth experience was significantly associated with childbirth self-efficacy (p = 0.005). Low self-efficacy was reported in study participants who did not receive preparation for their birth experience (n = 23, 26.4%), while high self-efficacy was reported in participants who were not prepared for their birth experience (n = 63, 72.41%).

Baseline characteristics of the intervention and control groups

An independent sample t-test indicated that, for childbirth self-efficacy, the inventory baselines of the intervention and control groups were equal. As shown in Table 5, the mean was 238.52 (SD = 56.79) for the control group and 237.45 (SD = 57.73) for the intervention group. Therefore, Leven’s test for assumed equal variances was insignificant (t(92) =.434, p =.665).

**Table 5:** Pretest Childbirth Self-Efficacy Difference Between Control and Intervention Groups

Pretest		Group	N	M		SD
	Childbirth self-efficacy	Control	48		238.5	56.79
		Intervention	46		237.4	57.73

Maternal self-efficacy paired measurement (pretest/posttest)

As shown in Table 6, there was a significant difference between the scores before the program (M = 238.00, SD = 56.94) and after (M = 259.27, SD = 48.39), with t (93) = -5.620, p = 0.00. A paired sample t-test was conducted to compare childbirth self-efficacy before and after the antenatal educational programs (Table 7).

**Table 6:** Mean Scores of Pretest and Posttest CBSEI

	N	Mean	SD
Childbirth self efficacy (pretest)	94	238.00	56.94
Childbirth self efficacy (posttest)	94	259.27	48.39

**Table 7:** Pretest/Posttest Childbirth Self-Efficacy Differences

Paired sample test						
Paired Differences		N	Mean	SD	df	P
Paired 1						
	Childbirth self-efficacy Pretest and posttest	94	9.13	14.44	93	.005
	Paired 2					
	Childbirth self-efficacy Pretest and posttest	94	21.27	36.70	93	.005

The effect of antenatal education on fear of childbirth and maternal self-efficacy

 As shown in Table 8, the posttest revealed a significant difference between the intervention group (who had received the antenatal education) and the control group (who had received only routine antenatal care), with a p <.05. The mean score for childbirth self-efficacy was significantly higher in the intervention group.

**Table 8:** Pretest/Posttest Maternal Self-Efficacy Differences

		Control Group (n = 48)		Intervention Group (n = 46)				
		M	SD	M	SD	P-Value		
Maternal self-efficacy	Pretest	238.52	56.79	237.45	57.73	.962		
	Posttest	242.97	53.29	276.28	35.96	.000*		

## Discussion

The intervention and control groups in this study had similar sociodemographic and obstetric characteristics. There were no significant differences in terms of the women’s ages, nationalities, residences, occupations, monthly incomes, educational levels, or support systems. Similarly, there were no significant differences between the intervention and control groups in relation to compliance with antenatal contact, number of antenatal contacts, sources of knowledge regarding labor and delivery, time spent with antenatal care providers, preparation for birth experience by prenatal providers, attendance on antenatal educational programs, and primary antenatal education received. In addition, no significant differences between the groups were revealed by the pretests on the CBSEI scale. This may be explained by the homogenous randomization and allocation-concealment method used during the distribution of the participants to the intervention and control groups. This decreased the possibility of baseline bias that could have influenced the group differences and may have affected the study results and outcomes. The study findings are consistent with those of the study conducted by Haapio et al. (2017), who examined the effects of childbirth education by midwives on the childbirth fears of ﬁrst-time mothers [19]. The study found that the participants in the control and intervention groups scored similarly for sociodemographic characteristics and obstetric data and in the pretest concerning fear of childbirth, which indicated successful randomization.

Maternal self-efficacy serves as an indicator of her ability to cope with labor and childbirth process [20]. According to Bandura (1977), self-efficacy is affected by one’s previous experiences. In the absence of previous experience, one is highly influenced by the experience, observations, and verbal persuasion of others [7]. In the present study, high self-efficacy was reported in participants aged 18-25 years. This may be explained by sociocultural factors that could have an impact on a pregnant woman’s view of childbirth. In contrast, a previous study by Schwartz et al. (2015) concludes that lower self-efficacy was reported by primiparous pregnant women but found no differences regarding the age of the participants [20]. Similarly, Salomonsson et al. (2013) concluded that there is no statistically significant link between maternal age and self-efficacy [21].

 The results show that housewives had significantly higher self-efficacy than participants who were employed or students. In contrast, Salomonsson et al. (2013) concluded that maternal occupation is not significantly associated with self-efficacy scores [21]. However, lower self-efficacy scores are strongly associated with higher scores for fear of childbirth [22]. Lower self-efficacy scores were also reported in participants who obtained their knowledge about labor and birth from family and friends, which may be explained by a lack of access to accurate information and negative childbirth stories leaving them more affected by the experiences of others. The result is in line with previous studies that have concluded lower self-efficacy is associated with fear and anxiety around childbirth [21,23,24]. Furthermore, the participants who were not prepared by their health care provider for their birth experiences reported lower self-efficacy scores. This may be explained by a lack of opportunity to discuss strategies for coping with the labor and childbirth process. This result is in line with those of previous studies concluding that preparation for birth enhances maternal self-efficacy [25,26].

In recent years, there has been controversy about the effects of antenatal educational programs on maternal self-efficacy. For instance, Gagnon and Sandal (2007) conducted a meta-analysis to assess the effect of these programs in relation to numerous variables, including maternal sense of control [10]. The results of this analysis were inconclusive. In contrast, Brixval et al. (2016) conducted a systemic review that revealed a significant and positive impact of antenatal education on maternal self-efficacy [16]. In the present study, the antenatal education program significantly enhanced maternal self-efficacy among primiparous pregnant women. The posttest showed significant improvements in self-efficacy among the intervention group compared to the control. This improvement may be attributed to the educational program in the present study overcoming one of the main contributors to lower self-efficacy, namely, a lack of support and preparation from the health care provider. In addition, the program in the current study aimed to provide the participants with the skills needed to cope with pain during childbirth, such as relaxation and breathing techniques, which act to improve self-confidence and enhance self-belief. In addition, the program in the current study aimed to provide the participants with the skills needed to cope with pain during childbirth, such as relaxation and breathing techniques, which act to improve self-confidence and enhance self-belief. This result is in line with a recent Egyptian randomized control trial which was conducted by El-Kurdy et al. (2017) [8]. In addition, the present study is in agreement with a Turkish study by Sercekus and Baskale (2016), which also used the CBSEI scale to investigate the effect of antenatal education on maternal self-efficacy. The pretest differences between the two groups were insignificant. However, the posttest scores for the intervention group (who had received antenatal education) were higher than those of the control group (who had received routine antenatal care) [24]. The results of the current study and those of many other studies emphasize the positive effects of antenatal education programs on self-efficacy. However, in contrast, Escott et al. (2005) found that childbirth preparation did not significantly enhance maternal self-efficacy and this variation may be due to relatively small sample sizes [27].

Implications for nursing and midwifery practice

The findings of this study suggest that primiparous Saudi women are in need of interventions to increase their self-efficacy. There is a need for investment in resources to empower and equip pregnant women with the information and skills they need, ensuring positive perceptions of pregnancy and labor.

Nurses and midwives can play an important role in designing and implementing structured antenatal educational programs as components of routine antenatal care in government hospitals. They can directly establish multidisciplinary teams that include a midwife, an obstetrician, and a psychotherapist trained to provide appropriate interventions and counseling for women with lower childbirth efficacy. Moreover, nurses and midwives can play an essential role in increasing awareness among pregnant women and their families of the need for education. It is recommended to establish reliable sources of information regarding pregnancy, labor, and childbirth, using various channels, such as the smartphone and social media, providing tools of reference for all antenatal women.

Study limitations

This study examined primiparous women; thus, the findings may not be applicable to multiparous women. Furthermore, a methodological limitation arises from the use of the posttest questionnaire: specifically, around 20% of the posttest questionnaires were conducted over the telephone, as the participants were unable to attend appointments with the researcher. In addition, this study was conducted at the National Guard Specialized Polyclinic in Jeddah, Saudi Arabia; therefore, the results may not be generalizable to other regions of the country.

## Conclusions

The findings of this study indicate that an antenatal educational program could serve as an essential tool, providing women with access to accurate information and skills during the antenatal period and thereby significantly enhancing their childbirth self-efficacy. Antenatal education is not currently part of the routine antenatal care in Saudi Arabia; therefore, pregnant women lack the opportunity to spend time with their health care providers, talking about pregnancy, labor, and the childbirth process and being prepared for their birth experience. The findings of the current study also indicate that women’s sociodemographic characteristics, obstetrical knowledge, and sources of knowledge are strongly linked to their level of childbirth self-efficacy.
